# Adherence to Posttreatment Surveillance Guidelines in Non–Small Cell Lung Cancer: Retrospective Cohort Study

**DOI:** 10.2196/76515

**Published:** 2025-10-01

**Authors:** Ryan J Randle, Scott V Adams, Zahra Esfahanimonfared, Nicole Lin, Julie Wu, Ann Leung, Steven M Asch, Steven Zeliadt, Alex Sox-Harris, Summer Han, Leah M Backhus

**Affiliations:** 1 Department of Cardiothoracic Surgery, Division of Thoracic Surgery, Stanford Medicine, 870 Quarry Rd, Stanford, CA, 94305, United States, 1 (650) 723-5771; 2 VA Puget Sound Health Care System, Seattle, WA, United States; 3 VA Palo Alto Health Care System, Palo Alto, CA, United States; 4 Department of Radiology, Stanford Medicine, Stanford, CA, United States; 5 Department of Medicine, Stanford Medicine, Stanford, CA, United States; 6 School of Public Health, University of Washington, , Seattle, WA, United States

**Keywords:** non–small cell lung cancer, surveillance, computed tomography imaging, CT imaging, survivorship

## Abstract

**Background:**

Several guidelines recommend posttreatment surveillance for non–small cell lung cancer (NSCLC). However, studies evaluating surveillance patterns often cannot distinguish between imaging ordered for surveillance versus for symptoms suggestive of recurrence. Moreover, early recurrences and other competing events hamper efforts to determine true surveillance rates because of wide variability in reported guideline adherence in clinical practice. Leveraging comprehensive Veterans Health Administration data, we developed a novel competing risks framework to describe the patterns and predictors of NSCLC imaging surveillance.

**Objective:**

This study aims to examine posttreatment surveillance to estimate the true surveillance rates and predictors of guideline-concordant care in patients with early-stage NSCLC.

**Methods:**

The study cohort comprised veterans who were treated for stage 1 to 3 NSCLC between 2008 and 2016 and who survived for ≥6 months. Clinical documents and radiology reports were abstracted for image indication and clinical information. We estimated the cumulative probability of receiving guideline-concordant surveillance, defined as chest computed tomography imaging within 4 to 9 months after treatment, accounting for competing risks and censoring. Multivariable cause-specific Cox regression was used to estimate associations between patient factors and guideline-concordant surveillance, with adjustments made for multiple comparisons.

**Results:**

The cohort consisted of 1888 patients. The mean age of the analysis cohort was 66.4 (SD 7.9) years; 95.9% (1811/1888) of the patients were male, 71.1% (1342/1888) of the patients were White, and 43.1% (814/1888) were married. Of the 1888 patients, 57% (n=1076) presented with stage 1 disease, and the most common treatment modality was surgery alone (n=1068, 56.6%). The most common type of imaging performed during the initial 120- to 270-day window was chest computed tomography (1460/3278, 44.5%). Chest X-rays accounted for 36.3% (1190/3278) of all imaging performed, while the remaining 11.8% (386/3278) and 7.4% (242/3278) were positron emission tomography scans or other imaging modalities, respectively. Compared to the years 2008 to 2010, patients treated for NSCLC from 2014 to 2016 had a significantly higher likelihood of receiving guideline-concordant surveillance (hazard ratio 1.42, *P*<.001).

**Conclusions:**

In this unique application of a competing risks framework, the rate of guideline-concordant surveillance in this national cohort was lower than that reported in many previous studies. This finding highlights a potentially substantial gap in surveillance among eligible, asymptomatic lung cancer survivors. More strategies are needed to measure the true rate of guideline-concordant surveillance, along with education and advocacy to ensure guideline-concordant care.

## Introduction

Lung cancer is one of the most common cancers worldwide and remains the leading cause of cancer mortality [1]. Following treatment for lung cancer, patients remain at risk both for recurrence and the development of second primary lung cancer (SPLC). Surgery remains the mainstay of curative-intent therapy in early-stage disease. However, patients can have recurrence rates ranging from 30% to 68% despite complete resection [2,3]. As a result, several expert bodies have issued guidelines recommending routine surveillance following treatment completion to detect recurrence and SPLC [4-6]. Nearly all these guidelines recommend imaging surveillance within 6 months following treatment completion.

Despite these recommendations for posttreatment surveillance, there is wide variability in guideline adherence in clinical practice. Previous studies have evaluated adherence to these recommendations for posttreatment surveillance, with reported rates ranging from 25% to 71% [7-10]. The cause of this variability remains unknown and is likely multifactorial, although differences in patient populations, demographics, ongoing access to specialized cancer care, and health care professional practice are likely factors [11]. In addition, the VA population faces unique stressors that may negatively influence health management behaviors, making them more vulnerable to stressors [12].

We contend that incomplete data on imaging indications are a potential contributing factor to the observed variability in guideline adherence. Imaging after definitive treatment can occur for multiple reasons, including nonsurveillance indications such as a chest computed tomography (CT) ordered for a traumatic injury. Previous studies that have included information on imaging indications have been limited to single-center experiences with a relatively small patient sample and are likely influenced by these population differences and varying institutional practices [8,13]. Large population-based studies that assess guideline-concordant surveillance across health care professional and patient characteristics have lacked information on imaging indications by relying on administrative claims–based data [7,9,10,14]. Patients may have imaging procedures captured in claims data despite competing events (ie, early recurrence) that exclude them from undergoing true surveillance, thereby leading to inaccurate estimates of posttreatment surveillance rates. Each of these limitations has hampered efforts to evaluate true surveillance rates and the effectiveness of surveillance programs more broadly.

Herein, this study examines posttreatment surveillance using detailed national Veterans Health Administration (VHA) data to estimate the true rate of adherent surveillance and predictors of guideline-concordant care in patients with early-stage non–small cell lung cancer (NSCLC). We used a novel annotation methodology to abstract imaging indications from national data at scale. Information on imaging indications and clinical documentation allowed us to use a competing risk analysis for estimating the cumulative probability of surveillance. In contrast to previously published reports, this form of time-to-event analysis offers the opportunity to account for events and describe the factors that affect posttreatment surveillance [15,16]. We expect that this analysis will provide a more accurate estimate of the rates and predictors of guideline-concordant posttreatment surveillance.

## Methods

### Cohort and Definitions

Using the VHA electronic health record databases, we identified 20,532 patients treated with curative intent for nonmetastatic primary NSCLC who were diagnosed between January 1, 2008, and April 30, 2016. We used the *International Classification of Diseases, 9th/10th Edition* (*ICD-9/10*) diagnosis codes (Table S1 in Multimedia Appendix 1). We also retrieved demographic, clinical, and vital status information for these patients. For inclusion, patients must have had either ≥1 inpatient or ≥2 outpatient NSCLC diagnosis codes, and the date of diagnosis must be the date of the earliest code demonstrating connectedness to care [17]. Curative-intent treatment was classified based on the *ICD-9/10* procedure codes and Current Procedural Terminology (CPT) codes (Table S2 in Multimedia Appendix 1) within 6 months of diagnosis. The NSCLC stage was classified according to the American Joint Committee on Cancer TNM (tumor, nodes, and metastasis) staging system (seventh edition in effect during the observation period). We excluded patients who did not receive curative-intent therapy, received chemotherapy only, died within 6 months of initiating therapy, had stage 4 cancer, had a non–skin cancer diagnosis (except melanoma and cervical cancer) within 5 years before the NSCLC diagnosis, or had any diagnosis of lung cancer before 2008. Participant follow-up ended on December 31, 2016.

The study cohort included a random subset of 1890 eligible patients with completely abstracted radiology reports and clinical notes from a 9238-sample cohort. An additional 369 patients from the sample cohort had no radiology reports directly relevant to NSCLC surveillance. To avoid overrepresentation of patients without abstractable radiology reports in our analysis, we weighted these patients so that their proportion in the study cohort was the same as their proportion among all eligible patients in the sample cohort—bringing the final analysis sample to 1888.

### Radiology Abstraction

We abstracted image indications (surveillance, symptoms, abnormality follow-up, other, and unknown) and modalities (positron emission tomography [PET]; chest X-ray [CXR]; chest CT; and other [magnetic resonance imaging, non–chest CT, and bone scan]) from radiology reports. Imaging indications were identified in the report text and recorded as documented.

We used the previously published semiautomated chart abstraction protocol by our group for obtaining detailed radiology data [18]. A searchable radiology index was created, which included full-text radiology reports that were then queried using SQL (structured query language) to identify all radiology-related text documents to be indexed. VISA, an open-source full-text search engine written in Java, was used to search the radiology reports for text relevant to the user query. Upon searching, VISA returned the user-defined full-text radiology reports with highlighted relevant text snippets, which were then manually reviewed, annotated, and coded by an abstractor.

### Clinical Abstraction

Clinical notes from patient charts were used to abstract any missing data on disease stage, smoking status, NSCLC recurrence or an SPLC, and tumor histology. Final pathologic staging took precedence during chart abstraction. Smoking at the time of diagnosis or initial presentation was considered “current.” The presence of recurrent NSCLC or SPLC was determined based on documentation by a treating clinician. Histologic subtypes were identified directly from pathologic reports when available or collected from other clinical note types (eg, progress notes). Patients who had small cell histology, had recurrence or SPLC outside the lungs within 120 days after treatment, or did not undergo curative-intent treatment were also excluded.

### Outcomes of Interest

The primary outcome was receipt of guideline-concordant surveillance imaging. The National Comprehensive Cancer Network (NCCN) recommendations for posttreatment surveillance imaging at the time of diagnosis were used as a base set of guidelines [19]. Although the published guidelines are updated, they have consistently recommended routine posttreatment surveillance with chest CT imaging approximately every 6 months during this study period. We defined guideline-concordant surveillance as performing chest CT imaging for surveillance purposes (as documented by the image indications) within 120 to 270 days following the first day of treatment or first day of last treatment with multimodality therapy. Imaging performed explicitly for surveillance purposes via other imaging modalities (eg, PET) was considered nonconcordant surveillance. The 120- to 270-day (4‐9 mo) posttreatment interval was adopted to represent the first surveillance window, with the intent of creating a more liberal window with a 2- to 3-month buffer on either end of the published 6-month guideline (Multimedia Appendix 1).

### Ethical Considerations

This study was approved by the institutional review boards of Stanford University and the Department of Veterans Affairs (IRB-46841). The study data used were deidentified. Informed consent was waived as per institutional review board review.

### Statistical Methods

To estimate the cumulative probability of patients receiving guideline-concordant surveillance imaging within the initial 120- to 270-day surveillance window after curative-intent therapy, we used a competing risks framework to account for competing events. This can help provide unbiased estimates of patients experiencing the event of interest, given that competing events materially alter their probability or preclude them entirely. For example, receiving a diagnosis of recurrent NSCLC before scheduled surveillance precludes guideline-concordant surveillance, as imaging after recurrence can no longer be considered surveillance. Recurrences discovered during surveillance imaging were coded as surveillance events. An example schematic of the time-to-event analysis helps visualize a few of these relatively common clinical scenarios (Figure S1 in Multimedia Appendix 1).

We used the Aalen-Johansen estimator and corresponding CIs to analyze the primary outcome, that is, the receipt of guideline-concordant surveillance imaging within the 120 to 270 days after curative-intent treatment. Death, recurrence, SPLC, and nonconcordant surveillance were treated as the competing events in this analysis.

To evaluate the factors associated with the primary outcome, we used multivariable cause-specific Cox regression to estimate hazard ratios (HRs) and CIs. We considered various demographic factors (eg, sex, age, race, marital status, and year of diagnosis) and clinical factors (eg, treatment, histology, and disease stage) in this model. To adjust for multiple testing, we applied the Bonferroni correction method for 11 independent variables in each model. We reported unadjusted *P* values and interpreted any value <.004 (<0.05/11) as significant. For consistency, we calculated 99.6% CIs for all point estimates. Missing data were treated as a separate category in models. To avoid very small groups, we combined stage 0 and stage 1 (due to the very small proportion of patients in stage 0). In addition, recurrences and SPLC malignancies were combined as a single competing event referred to as “recurrence” for brevity. A univariable model of the primary outcome is shown in Table S4 in Multimedia Appendix 1. Statistical analysis was performed using R (version 4.2.2) [20].

## Results

### Demographics of the Analytic Cohort

**Figure 1.**
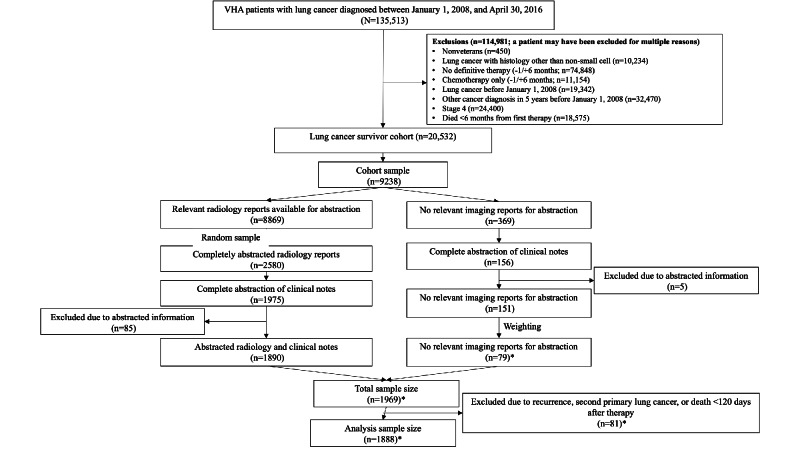
CONSORT (Consolidated Standards of Reporting Trials) flowchart. VHA: Veterans Health Administration.

Of 135,513 patients diagnosed with primary NSCLC between January 1, 2008, and April 30, 2016, a total of 20,532 met the study eligibility requirements (Figure 1). The study cohort comprised a random sample of 1890 patients with abstracted clinical notes and radiology reports, while 369 of all eligible patients had no relevant radiology reports available; these 369 patients were weighted to prevent overestimation. The effective study sample size was 1888 patients after weighting (see *Cohort and Definitions* section; Table 1). The mean age of the analysis cohort was 66.4 (SD 7.9) years, of which 95.9% (1811/1888) were male, 71.1% (1342/1888) were White, and 43.1% (814/1888) were married. Of the 1888 patients, 57% (n=1076) presented with stage 1 disease, and the most common treatment modality was surgery alone (n=1068, 56.6%).

**Table 1.** Participant characteristics at the time of diagnosis (weighted; n=1888).

	Participants
Diagnosis year, n (%)	
2008‐2010	628 (33.3)
2011‐2013	726 (38.5)
2014‐2016	534 (28.3)
Age (mean 66.4, SD 7.9 y), n (%)	
<65	817 (43.3)
65‐74	746 (39.5)
≥75	324 (17.2)
Sex (male), n (%)	1811 (95.9)
Race, n (%)	
Black or African American	327 (17.3)
Other	105 (5.5)
White	1342 (71.1)
Missing	114 (6.1)
Marital status (married), n (%)	814 (43.1)
Region, n (%)	
Mid-Atlantic	376 (19.9)
Midwest	238 (12.6)
Northeast	191 (10.1)
South	743 (39.3)
West	339 (18)
Smoking status, n (%)	
Current	947 (50.1)
Former	591 (31.3)
Never	316 (16.8)
Missing	34 (1.8)
Charlson Comorbidity Index, n (%)	
0	539 (28.6)
1	597 (31.6)
>1	752 (39.8)
Disease stage, n (%)	
0 or 1	1076 (57)
2	329 (17.4)
3	414 (21.9)
Missing	69 (3.6)
Histology, n (%)	
Adenocarcinoma	876 (46.4)
Squamous and transitional cell	753 (39.9)
Other	196 (10.4)
Missing	64 (3.4)
Initial therapy, n (%)	
Surgery alone	1068 (56.6)
Radiation and chemotherapy	251 (13.3)
Surgery and chemotherapy	235 (12.5)
Radiation alone	181 (9.6)
Surgery, chemotherapy, and radiation	95 (5)
Surgery and radiation	57 (3)

### Distribution of Imaging Performed

Overall, the most common imaging performed during the initial 120- to 270-day window was chest CT (1460/3278, 44.5%; Table 2). CXR accounted for 36.3% (1190/3278) of all imaging performed, while the remaining 11.8% (386/3278) and 7.4% (242/3278) were PET scans or other imaging modalities, respectively. Chest CT was found to be the most common modality when evaluating imaging performed for surveillance. The proportion of CXR imaging performed for surveillance was 12.2% (176/1448), while the proportion of chest CT and PET imaging performed for surveillance was 70.9% (1026/1448) and 14.4% (209/1448), respectively. Chest CT remained the most common imaging procedure for surveillance across all types of primary treatment groups (Table 3). However, PET imaging comprised 24% (46/192) of surveillance imaging in patients receiving radiation therapy as a part of their primary treatment (38/129, 29.5% in radiation alone), compared to 14.4% (209/1448) across all other treatment groups combined.

**Table 2.** Imaging performed during the 120- to 270-day surveillance window by indication and modality^a^.

	Chest CT^b^ (n=1460), n (%)^c^	CXR^d^ (n=1190), n (%)^c^	PET^e^ (n=386), n (%)^c^	Other^f^ (n=242), n (%)^c^	Total (n=3278), n (%)^g^
Indication					
Surveillance	1026 (70.9)	176 (12.2)	209 (14.4)	37 (2.6)	1448 (44.1)
Symptoms	88 (15.7)	357 (63.5)	8 (1.4)	109 (19.4)	562 (17.1)
Follow-up	117 (39.3)	46 (15.4)	109 (36.6)	26 (8.7)	298 (9)
Other or unknown	229 (23.6)	611 (63)	60 (6.2)	70 (7.2)	970 (29.5)

a Patients may be counted in more than one column if they underwent multiple imaging procedures.

b CT: computed tomography.

c Percentages in this column were calculated by using the row total as the denominator.

d CXR: chest X-ray.

e PET: positron emission tomography.

f Other: magnetic resonance imaging, nonchest CT, and bone scan.

g Percentages in this column were calculated by using the column total as the denominator.

**Table 3.** Imaging performed for surveillance during the 120- to 270-day interval by therapy and modality^a^.

	Chest CT^b^ (n=1026), n (%)^c^	CXR^d^ (n=176), n (%)^c^	PET^e^ (n=209), n (%)^c^	Other^f^ (n=37), n (%)^c^	Total (n=1448), n (%)^g^
Therapy					
Surgery alone	606 (75.3)	119 (14.8)	58 (7.2)	22 (2.7)	805 (55.5)
Radiation and chemotherapy	128 (66.7)	12 (6.2)	46 (24)	6 (3.1)	192 (13.2)
Surgery and chemotherapy	130 (68.1)	21 (11)	34 (17.8)	6 (3.1)	191 (13.1)
Radiation alone	78 (60.5)	12 (9.3)	38 (29.5)	1 (0.8)	129 (8.9)
Surgery, chemotherapy, and radiation	57 (68.7)	6 (7.2)	20 (24.1)	0 (0)	83 (5.7)
Surgery and radiation	27 (56.2)	6 (12.5)	13 (27.1)	2 (4.2)	48 (3.3)

a Patients may be counted in more than one column if they underwent multiple imaging procedures.

b CT: computed tomography.

c Percentages in this column were calculated by using the row total as the denominator.

d CXR: chest X-ray.

e PET: positron emission tomography.

f Other: magnetic resonance imaging, nonchest CT, and bone scan.

g Percentages in this column were calculated by using the column total as the denominator.

### Cumulative Probability of Receiving Posttreatment Imaging

**Figure 2.**
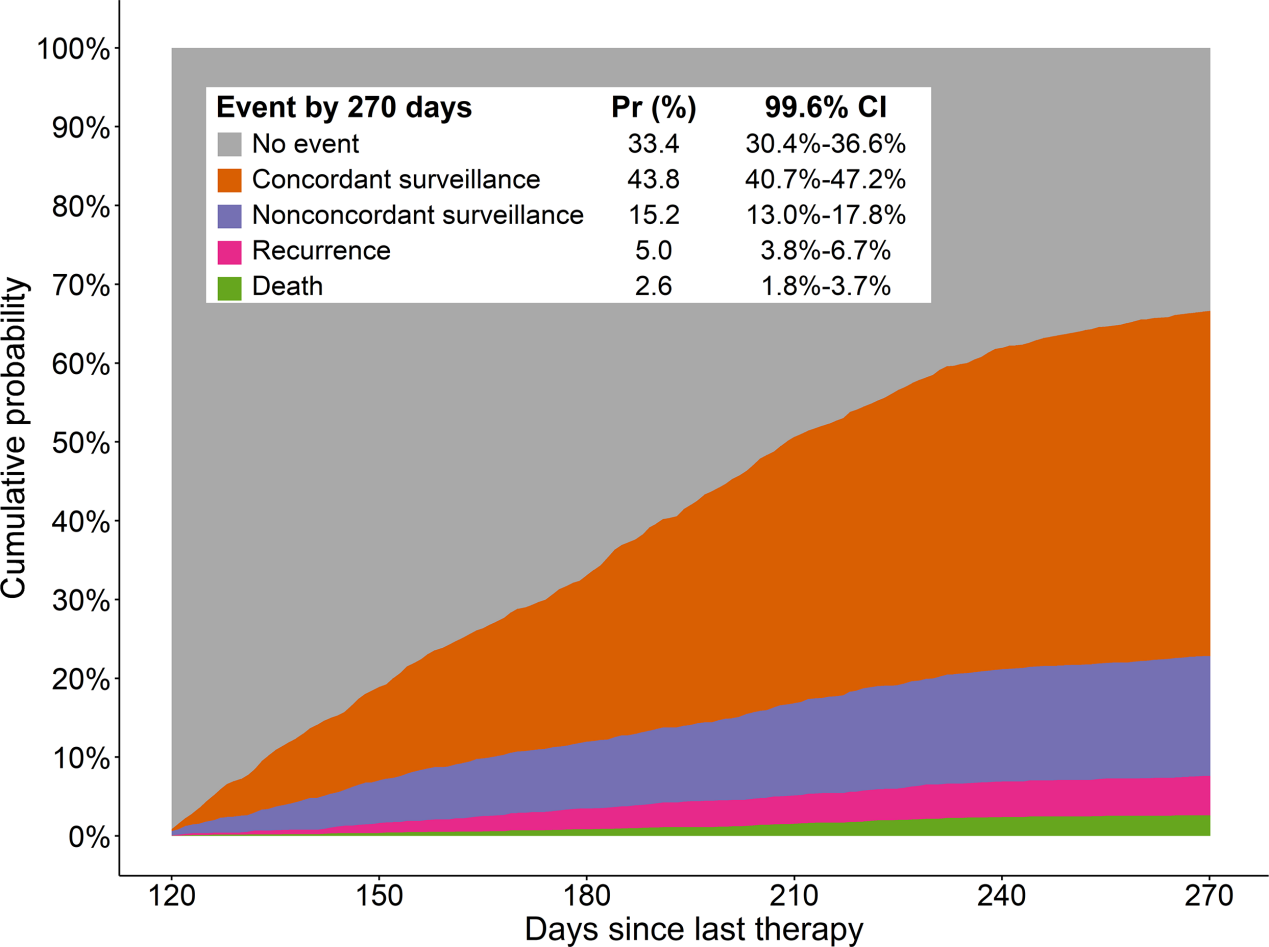
Cumulative probability estimated from an Aalen-Johansen competing events model for receipt of guideline-concordant imaging, defined as chest computed tomography imaging with a documented indication of surveillance, within 120 to 270 days after definitive therapy. The probability curves are stacked such that the sum at any time point is 100%. Patients were followed only until the first event, and cumulative probability is therefore the expected proportion of patients who had the event as their first event before a given time point. The overlaid table shows the cumulative probability (Pr) and 99.6% CI at 270 days after therapy.

To estimate the cumulative probability of patients receiving guideline-concordant surveillance imaging after curative-intent therapy, we used a competing risks framework to correctly estimate the cumulative probability by accounting for competing events (eg, death or recurrence). This analysis shows that the cumulative probability of receiving guideline-concordant imaging for surveillance was 43.8% (827/1888; 99.6% CI 41.6%-46.1%; Figure 2). The cumulative probability of receiving nonconcordant surveillance imaging within this window (120-270 days) was 15.2% (287/1888; 99.6% CI 13.0%-17.8%), whereas 33.4% (630/1888; 99.6% CI 30.4%-36.6%) of patients received no imaging at all. A total of 5% (95/1888; 99.6% CI 3.8%-6.7%) of patients experienced recurrence, while 2.6% (49/1888; 99.6% CI 1.8%-3.7%) of patients died during the posttreatment surveillance period, that is, roughly 7.6% (144/1888) of patients were ineligible for surveillance during this period.

### Factors Associated With Receiving Guideline-Concordant Surveillance Imaging

We evaluated the association between the receipt of guideline-concordant surveillance imaging and various demographic and clinical variables using multivariable cause-specific Cox regression (Table 4). Compared to patients treated for NSCLC between 2008 and 2010, patients receiving treatment from 2014 to 2016 were significantly more likely to receive guideline-concordant surveillance imaging (HR 1.42, 99.6% CI 1.09-1.87, *P*<.001). There was a similar trend toward an increased likelihood of guideline-concordant surveillance in years 2011 to 2013 compared to 2008 to 2010, although this did not reach statistical significance. No other variables were significantly associated with receipt of guideline-concordant surveillance imaging. Although radiation alone was not significantly associated with a decreased likelihood of receiving guideline-concordant surveillance imaging (HR 0.68, 99.6% CI 0.45-1.03, *P*=.007), radiation alone was associated with an increased likelihood of nonconcordant surveillance imaging in the same model (HR 1.93, 99.6% CI 1.10-3.38, *P*<.001) consistent with the increased use of PET for surveillance in this group (Table S3 in Multimedia Appendix 1).

**Table 4.** Results of multivariable cause-specific Cox regression for receipt of guideline-concordant surveillance imaging, defined as chest computed tomography with a documented indication of surveillance, within 120 to 270 days of definitive therapy; competing events were defined as the earliest occurrence of recurrence or second primary lung cancer, death, or nonconcordant surveillance imaging.

	Concordant surveillance	
	HR^a^ (99.6% CI)	*P* value^b^
Therapy		
Surgery alone	Reference	—^c^
Surgery and chemotherapy	1.08 (0.76-1.54)	.53
Surgery and radiation	1.10 (0.60-2.01)	.66
Surgery, chemotherapy, and radiation	1.07 (0.64-1.79)	.72
Radiation alone	0.68 (0.45-1.03)	.007
Radiation and chemotherapy	0.93 (0.61-1.42)	.61
Disease stage		
0 or 1	Reference	—
2	0.77 (0.56-1.06)	.02
3	0.91 (0.65-1.29)	.44
Missing	0.42 (0.17-1.01)	.005
Diagnosis year		
2008‐2010	Reference	—
2011‐2013	1.26 (0.98-1.62)	.009
2014‐2016	1.42 (1.09-1.87)	<.001^d^
Age (y)		
<65	Reference	—
65‐74	1.06 (0.84-1.34)	.50
>75	1.17 (0.86-1.59)	.15
Sex (male vs female)	1.46 (0.81-2.62)	.06
Histology		
Adenocarcinoma	Reference	—
Squamous and transitional cell	0.95 (0.76-1.18)	.49
Other	1.03 (0.73-1.45)	.82
Missing	0.99 (0.45-2.19)	.97
Marital status (married vs unmarried)	1.11 (0.90-1.37)	.14
Race		
Black	Reference	—
White	1.01 (0.77-1.34)	.90
Other	1.19 (0.74-1.91)	.30
Missing	0.78 (0.46-1.32)	.17
Region		
Mid-Atlantic	Reference	—
Midwest	0.98 (0.69-1.39)	.85
Northeast	1.41 (1.00-2.00)	.004
South	0.77 (0.57-1.02)	.008
West	0.98 (0.71-1.36)	.88
Smoking		
Current	Reference	—
Former	0.94 (0.75-1.20)	.48
Never	0.79 (0.58-1.07)	.02
Missing	0.92 (0.44-1.91)	.73
Charlson Comorbidity Index		
0	Reference	—
1	0.91 (0.70-1.17)	.28
>1	0.99 (0.77-1.27)	.92

a HR: hazard ratio.

b To account for multiple hypothesis tests, *P*<.004 was interpreted as statistically significant and 99.6% CIs were estimated; the displayed *P* values were not adjusted.

c Not available.

d *P*<.004.

### Factors Associated With Recurrent Cancer or SPLC and Death

There were no significant factors associated with developing recurrence or SPLC in the multivariable cause-specific Cox proportional hazards model for competing outcomes (Table S3 in Multimedia Appendix 1). Compared to surgery alone, treatment with surgery and radiation was associated with an increased likelihood of death in the same model (HR 12.3, 99.6% CI 2.52-60.19, *P*<.001).

## Discussion

### Principal Findings

Despite widespread recommendations for posttreatment surveillance, we found that roughly one-third of patients undergoing treatment for NSCLC before 2016 did not receive any form of surveillance. Moreover, the true fraction of patients receiving surveillance imaging consistent with recommended guidelines in this study was less than half (43.8%). This is an improvement over results from our previous study of patients’ first imaging procedures within 4 to 8 months after resection (representing the first surveillance window) of early-stage NSCLC in the Medicare population, where 24.6% of patients received a chest CT scan and 59.6% received a CXR [7]. Similar to other studies, our work demonstrates evidence of the increasing use of posttreatment chest CT imaging for surveillance over time in line with routine surveillance guidelines [9,14]. Sustained improvement in posttreatment surveillance adherence necessitates dedicated efforts both at the individual level, through education and interventions, and at a systems level, through multidisciplinary collaboration and advocacy.

### Comparison to Existing Literature

We observed that the rate of guideline-concordant surveillance CT imaging is substantially lower than that previously reported by Malhotra et al [10] and Erb et al [9]. In their 2018 study using SEER-Medicare data, Malhotra et al [10] defined adherent imaging as a patient receiving at least a CT scan or PET and CT scan during the 7- to 18-month, 19- to 30-month, 31- to 42-month, and 43- to 60-month intervals after treatment. The rate of adherent surveillance imaging at 18 months was 70.6%. The variation in reported rates across these and other studies is likely reflected in the fact that very few large studies concerning posttreatment surveillance contain information on the indication for posttreatment imaging. Therefore, many of these studies likely reflect imaging done for various indications, including the evaluation of symptoms (eg, cough and pneumonia), or are unknown and do not truly reflect imaging done for the explicit purpose of surveillance. In addition, the study by Malhotra et al [10] defines the receipt of adherent imaging as receiving CT or PET and CT despite guideline recommendations against the routine use of PET imaging for posttreatment surveillance of NSCLC, which was excluded in our definition. Our study, as well as other studies, has demonstrated that nonconcordant surveillance imaging makes up a significant fraction of radiology evaluations in patients treated for NSCLC. This may be due in part to clinical practice variations in using distinct treatment modalities to monitor treatment response or disease progression (ie, the use of PET or CT imaging in patients undergoing radiation or neoadjuvant therapy). Differences in patient population, comorbidities, and systems of care delivery also likely play some role in varying the reported rates of surveillance.

### Strengths

An important strength of our study is the inclusion of detailed VHA data that provided insights into the indications for radiology procedures. Indeed, true rates of posttreatment surveillance were previously obscured by the fact that imaging reported from other studies may have been done for purposes other than surveillance, as it is nearly impossible to reliably categorize imaging indications using administrative and claims data alone. Another strength of our study was the ability of the competing risks model to evaluate true surveillance rates in the presence of competing events that alter estimates put forth in other studies. Indeed, we found 7.6% (144/1888) of patients were not eligible to receive surveillance in our sample cohort because of death or recurrence. Accounting for competing events addresses the potential for overestimating the incidence of the event of interest, a potential weakness of using the Kaplan-Meier method or claims-based data in this setting. Each of these features present in this study likely describes our report of relatively lower rates of guideline-concordant surveillance compared with the literature. Thus, using the most liberal interpretation of our data, excluding those patients with competing events of early recurrence or death, 59% (1114/1888) of our cohort received imaging with the explicit intent of surveillance (44%, 827/1888 guideline concordant and 15%, 287 discordant). This is consistent with other reports lacking indication data, yet 33% (630/1888) received no imaging at all during this initial surveillance period. This allows us to highlight the latter group of patients with no surveillance imaging as the most important group to target when considering interventions in the future.

### Limitations

There are limitations to be acknowledged in this study. First is the use of administrative *ICD-9/10* and CPT codes to identify patients with relevant treatment procedures. We did not have structured diagnosis dates and staging information from a cancer registry for cohort construction. Thus, we used a conservative approach to ensure patients included in the cohort were newly diagnosed with NSCLC. Our approach may have missed some patients with a previous NSCLC diagnosis and the 20,532-patient cohort such that the data may not reflect the total incidence of early-stage NSCLC in VHA data during this period. Second, we abstracted only radiology report information on patients receiving imaging studies at VHA facilities. Veterans who received relevant imaging studies at external facilities do not have reports that are easily viewable in the VHA database except through an archival process requiring a review request of any scanned reports that were provided by the community imaging clinic. In our sensitivity analyses, we examined the rates of non-VA care including imaging paid for by the VHA (fee-based care) and care that veterans received through Medicare or Medicaid. Data are not available for any community care services patients pay for out of pocket or those covered by other insurance providers such as a veteran’s private insurance provider, although dual use of care from private insurance providers is rare among patients who otherwise rely on the VHA for the majority of their care and, in particular, cancer care [21]. This sensitivity analysis identified that among the entire cohort, fewer than 16% of veterans had any non-VA imaging scans performed in the available administrative data, with 8% of these scans corresponding to CT scans. Thus, the inclusion of these studies would still result in the underuse of surveillance overall. Third, our dataset reflects patterns from 2008 to 2016 as well as care within the VHA, which is a more homogenous population than the general population. Given the changing guidelines, practice patterns, and differences in the general population, our observations may differ from the reality of the non-VA population in the current state. Yet, cancer outcomes within the VHA and quality of care (including lung cancer in particular) have been reported in the literature as being noninferior and at times superior to lung cancer care provided in the non-VA population [22]. Thus, if anything, our main limitation might be in underestimating the true adherence rate in the non-VA setting.

### Future Directions

In conclusion, the use of this detailed clinical database, along with the novel application of a competing risks model, has provided additional clarity on the current state of adherence to posttreatment surveillance. Our analysis shows that the rate of guideline-concordant surveillance in this national cohort was lower than that reported in many studies. There is a need to develop efficient strategies to monitor the rate of guideline-concordant surveillance as well as patient education and health care professional advocacy to ensure these patients do not fall through the cracks of the health system. In addition, future studies are needed to determine how guideline adherence and other aspects of current surveillance practices influence clinical outcomes, especially given the recent literature casting doubts on the survival benefits of routine surveillance imaging [22,23].

## Supplementary material

10.2196/76515 Multimedia Appendix 1 Example schematic of the time-to-event analysis with competing risks. The multicolored banners represent individual models with the event of interest and their corresponding competing risks. Each horizontal arrow is a single hypothetical patient undergoing review within the 120- to 270-day posttreatment period.

## Abbreviations:

CPT: Current Procedural Terminology
CT: computed tomography
CXR: chest X-ray
HR: hazard ratio
*ICD-9/10*: *International Classification of Diseases, 9th/10th Edition*
NCCN: National Comprehensive Cancer Network
NSCLC: non–small cell lung cancer
PET: positron emission tomography
SPLC: second primary lung cancer
SQL: structured query language
TNM: tumor, nodes, and metastasis
VHA: Veterans Health Administration
