# Supplementary figures and images for: Adherence to Posttreatment Surveillance Guidelines in Non–Small Cell Lung Cancer: Retrospective Cohort Study
Source: JMIR Cancer. 2025 Oct 1;11:e76515. doi: 10.2196/76515 (PMC12488033; doi:10.2196/76515)

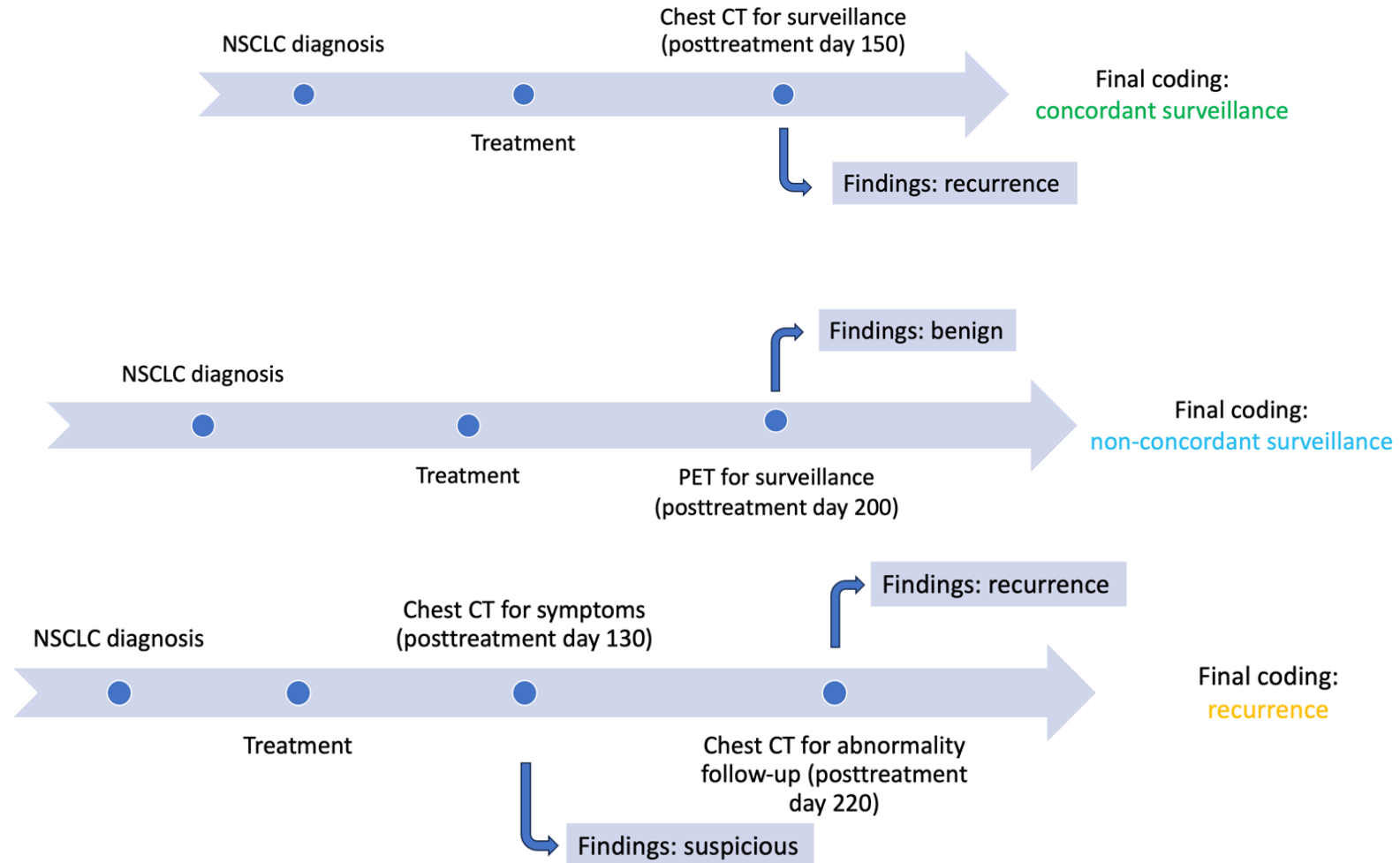

Events: no event, concordant surveillance, non-concordant surveillance, recurrence, death

Supplement: Multimedia Appendix 1 [file cancer-v11-e76515-s001.pdf]
